# Meta-analysis of the literature on diagnostic accuracy of SPECT in parkinsonian syndromes

**DOI:** 10.1186/1471-2377-7-27

**Published:** 2007-09-01

**Authors:** Annemarie MM Vlaar, Marinus JPG van Kroonenburgh, Alfons GH Kessels, Wim EJ Weber

**Affiliations:** 1 Department of Neurology, University Hospital Maastricht, The Netherlands; 2 Department of Nuclear Medicine, University Hospital Maastricht, The Netherlands; 3 Department of Clinical Epidemiology and Technology Assessment, University Hospital Maastricht, The Netherlands

## Abstract

**Background:**

Parkinson's disease (PD) is the second most common neurodegenerative disorder. One of the most widely used techniques to diagnose PD is a Single Photon Emission Computer Tomography (SPECT) scan to visualise the integrity of the dopaminergic pathways in the brain. Despite this there remains some discussion on the value of SPECT in the differential diagnosis of PD. We did a meta-analysis of all the existing literature on the diagnostic accuracy of both pre- and post-synaptic SPECT imaging in the differential diagnosis of PD.

**Methods:**

Relevant studies were searched in Medline, EMBASE and Cochrane databases with back-searching of their reference lists. We limited our analysis to studies with a clinically relevant methodology: i.e. when they assessed the ability of the SPECT to provide 1. diagnosis of PD in an early phase vs. normalcy; 2 diagnostic differentiation between PD and essential tremor (ET); 3. distinguishing between PD and vascular parkinsonism (VP); 4. delineation of PD from atypical parkinsonian syndromes (APS). Gold standard was, dependent on the study type, clinical examination at initial visit or follow-up, and/or response to dopaminergic agents.

**Results:**

The search gave 185 hits, of which we deemed 32 suitable for our analysis. From these we recalculated the diagnostic odds ratio of SPECT for the clinical questions above. The pooled odds ratio (with 95%CI) for presynaptic SPECT scan's ability to distinguish between early PD and normalcy was 60 (13 – 277). For the ability to differentiate between PD and ET this ratio was 210 (79–562). The ratio for presynaptic SPECT's ability to delineate PD from VP was 105 (32 – 348). The mean odds ratio for the presynaptic SPECT scans to differentiate between PD and the two APS was 2 (1 – 4), and for the postsynaptic SPECT imaging this was 19 (9–36).

**Conclusion:**

SPECT with presynaptic radiotracers is relatively accurate to differentiate patients with PD in an early phase from normalcy, patients with PD from those with ET, and PD from VP.

The accuracy of SPECT with both presynaptic and postsynaptic tracers to differentiate between PD and APS is relatively low.

## Background

Parkinson's disease (PD) is the second most common neurodegenerative disorder with a life-time risk of 2 percent in males and 1.3 percent in females [1]. In most cases the diagnosis of PD is straightforward when cardinal clinical signs and symptoms as bradykinesia, rigidity, and resting tremor are present [2]. However, these main features of PD are shared, at least in part, by essential tremor (ET), multisystem atrophy (MSA), progressive supranuclear palsy (PSP), vascular parkinsonism (VP), dementia with Lewy bodies, corticobasal degeneration, Alzheimer's disease, and drug-induced parkinsonism. Besides delineating PD from the above parkinsonian disorders, distinguishing PD from normality can also be difficult, especially in early stage of the disease [3].

The gold standard for the diagnosis of PD is post-mortem neuropathological examination [2,4]. Neuropathological studies show that even at end-stage disease the clinical diagnostic accuracy for PD varies between 75–90%, with MSA and PSP accounting for most false positives [2,5-7]. Diagnostic accuracy is certainly less than 90% in earlier disease, as Litvan et al. found that the median sensitivity for the diagnosis of PD increased from 73% at the first visit to 80% to the last visit after a mean follow-up of 9 years, and the median positive predictive value increased from 46 to 64% [8].

A reliable test to diagnose PD is important for two reasons. Prognosis and management of PD and other parkinsonian disorders differ considerably [9], and an objective disease marker would facilitate the development of neuroprotective therapies [10]. Several procedures have been proposed to diagnose PD: functional imaging with Positron Emission Tomography (PET) or Single Photon Emission Computer Tomography (SPECT), transcranial sonography, olfactory- and neuropsychological tests, biomarkers and DNA tests [11-14].

At the moment neuro-imaging techniques like PET and SPECT are the most widely used diagnostic tools [9]. PET is at least as reliable as SPECT, but its use in routine clinical practice is limited by high costs and a relative short half-life of its radioactive tracers [15-18]. Different radiotracers can be used to visualise the nigrostriatal system. Presynaptic tracers are used to visualize the dopamine transporter, and postsynaptic radiotracers to assess dopamine receptor density. Examples of presynaptic radiotracers are Iodine-123-beta-CIT, Iodine-123-Ioflupane (FP-CIT), Iodine-123-IPT and 99mCT-TRODAT-1. Examples of postsynaptic tracers are Iodine-123-iodobenzamide and Iodine-123-Iodobenzofuran [19].

Despite its widespread use, there is no consensus about the value of SPECT in the differential diagnosis of PD. First, comparisons between this functional dopaminergic imaging and the ultimate gold standard, autopsy-proven PD, are almost non-existent [20,21]. Second, using a surrogate gold standard in the form of a long-term clinical follow-up, the ability of SPECT to discriminate PD from normality and/or other parkinsonian disorders and to distinguish one of the atypical parkinsonian syndromes from the other varies greatly among different studies. Comparing these studies is difficult, as they use different radiotracers and SPECT techniques, and, more importantly, involve different patient populations. A major issue here is that many studies use clearly-defined later-stage patients that are obviously not representative for the diagnostic problem that one wants to solve with a SPECT.

With this perspective of clinical relevance we did a meta-analysis of all the existing literature on the diagnostic accuracy of both pre- and post-synaptic SPECT in the differential diagnosis of PD. We defined studies as clinically relevant when they dealt with the ability of the SPECT to identify PD in patients with diagnostic uncertainty, to delineate PD from the other parkinsonian disorders and ET, and to provide an early diagnosis of PD in patients with little signs and symptoms.

## Methods

### Data sources

Meta-analysis was done according to current methodological recommendations [22-24]. We searched MEDLINE using the following terms: PD, parkinsonian, MSA, PSP, VP and ET. We searched for MeSH terms and free text words. All in combination with SPECT and clinical trial. No beginning data limit was used. The search was updated until 9 January 2006. Only English-, Dutch- and German language studies were considered, because the investigators were familiar with these languages. The bibliographies of selected articles were screened for potentially suitable references which were then retrieved. We also searched the EMBASE and Cochrane database (Wiley InterScience) using the same search strategy.

### Study selection

Two investigators (AV, WW) screened the full text of potential relevant articles using the inclusion criteria. For this we use a standard form combined with a modified QUADAS score, see table 1 (form available upon request) [25]. In all cases the investigators reached consensus. Studies were selected if the subject of the study was in one of the following three areas:

**Table 1:** Methodological aspects of all included trials.

Author	Number of patients (exclusive controls) (1)	Study methodology (2)	Patiënt selection consequetive	Golden standard (3)	Clinical daignostic criteria clearly described?	Minimal duration follow-up after scan (month)	Radiotracer: pre- or postsynaptic or both	Name of radiotracer (4)	SPECT judged visually, template or drawn (5)	Part of striatum judged (6)	Cut-off point of 2 sd's taken by the authors?	Drug stopped appropriate before SPECT? (7)	SPECT judged blindly for clinical diagnoses?
Asenbaum '98	61	II, III	-	cc	yes	-	pre	beta	m	striatum	2sd	yes	yes
Benamer '00	185	II	-	cc	yes	-	pre	fpcit	t	striatum	-	yes	yes
Booij '01	20	I	-	cf	yes	24	pre	fpcit	t	striatum	2sd	-	yes
Buck '95	23	II	-	cc	yes	-	post	ibf	t	striatum	2sd	-	-
Eerola '05	135	I	yes	cf	yes	24	pre	beta	m	striatum	-	yes	-
Gerschlager '02	33	II	-	cc	yes	-	pre	beta	m	striatum	-	yes	-
Haapaniemi '01	29	III	-	cf	yes	24	pre	beta	t	striatum	-	yes	yes
Huang '01	34	III	-	cc	yes	-	pre	beta	m	putamen	-	yes	-
Kim '02	31	II	-	cc	yes	-	both	beta/ibf	t	c-putamen	-	yes	yes
Laere V '04	62	III	yes	.	-	.	pre	fp/tr	t	c-putamen	-	yes	-
Lee '99	26	II	-	cc	yes	-	pre	ipt	m	striatum	-	yes	-
Lokkegaard '02	72	I	yes	cf	yes	14	pre	beta	t	striatum	-	-	yes
Lu '04	85	II	-	cc	yes	-	pre	trodat	t	c-putamen	-	-	yes
Messa '98	18	II	-	cc	yes	-	pre	beta	m	c-pc	-	-	-
Muller '98	24	III	-	dd	yes	-	pre	beta	m	striatum	-	yes	yes
Oertel '93	67	II	-	fd	-	3	post	ibzm	t	striatum	2sd	yes	-
Oyanagi '02	13	II	-	cc	yes	-	post	ibf	t	striatum	-	yes	-
Pirker '97	19	II	-	cc	yes	-	post	epide	m	striatum	-	yes	-
Pirker '00	78	II	-	cc	yes	-	pre	beta	m	striatum	-	yes	-
Pirker '02	51	II	-	cc	yes	-	pre	beta	m	striatum	-	yes	yes
Plotkin '05	57	II	yes	cc	yes	-	both	fp/ibzm	t	putamen	-	yes	yes
Rooyen v '93	21	II	-	cc	-	-	posr	ibzm	m	striatum	-	yes	-
Schelvsky '93	44	II	yes	dd	-	-	post	ibzm	t	striatum	-	-	-
Schwarz '98	65	I	-	fd	yes	24	post	ibzm	t	striatum	2sd	yes	yes
Schwarz '94	20	II	-	cc	-	-	post	ibzm	t	striatum	-	-	-
Schwarz '00	28	III	-	dd	yes	-	pre	ipt	t	striatum	-	-	-
Schwarz '97	55	I	-	fd	-	24	post	ibzm	t	striatum	2sd	-	yes
Schwarz '93	62	I	yes	fd	-	4	post	ibzm	m	striatum	2sd	yes	yes
Seppi '04	32	II	yes	cc	yes	-	post	ibzm	m	striatum	2sd	yes	-
Stoffers '05	70	I	-	cf	yes	36	pre	beta	t	c-pc	2sd	yes	-
Tatsch '91	42	II	-	cc	-	-	post	ibzm	t	striatum	2sd	yes	-
Vlaar '06	147	I	yes	cf	yes	3	both	fp/ibzm	t	putamen	2sd	yes	yes

- means "no" or information not clearly mentioned by the authors

1) Number of patients of particular studied included in meta-analysis. Healthy controls and patients with diseases not relevant or inconclusive diagnosis for this study are not counted.

2) Study methodology

I = patients with diagnostic uncertainty. Diagnostic accuracy of SPECT was determined with a surrogate gold standard

II = cross sectional study of already diagnosed patient categories

III = early PD vs. normalcy

3) cf = clinical criteria after follow-up

cc = clinical criteria without follow-up

dd = effect dopaminergic drugs or apomorphine test

fd = clinical criteria after follow-up & effect dopaminergic drugs or apomorphine test

4) beta = Iodine-123-beta-CIT (beta-CIT), fpcit = Iodine-123-Ioflupane (FP-CIT), tr = Iodine-123-IPT and 99mCT-TRODAT-1.

ibzm = 123I-iodobenzamide (IBZM), ibf = Iodine-123-Iodobenzofuran (IBF), epide = Iodine-123-epidepride.

5) t = region of interest determined with template

m = region of interest manually encircled

6) bg = basale ganglia or striatum, put = putamen, cp = contralateral putamen, cpc = contralateral putamen/caudate ratio

7) Yes if: drugs with interference with radiotracer were stopped appropriately or if the subjects did not use dopaminergic drugs at the moment of the spect.

(-) if this information was not given or if dopaminergic drugs seemed not to be stopped appropriately.

1. Patients who underwent SPECT because of diagnostic uncertainty.

2. Cross-sectional study of already diagnosed patient categories, in which SPECT was tested as a means to differentiate between various parkinsonian syndromes.

3. Cross-sectional studies with patients with known PD in an early stage (Hoehn & Yahr stage 2 or less) vs. normal healthy controls, in which SPECT was tested as a means to provide an early diagnosis.

### Exclusion criteria

The following exclusion criteria were used: 1) whole article not available, 2) language different from English, German or Dutch, 3) studies including only advanced PD patients vs. healthy controls, and studies with other main categories, e.g. dementia, 4) study population with less than 10 patients, 5) if the numbers of true positives, false negatives, true negatives and false positives with a cut-off point of 2 standard deviations (SD) from the mean of the control group were not available or could not be derived the study was excluded.

When the study included more than 85 patients we contacted the corresponding author to ask for the raw data (see below).

We chose this approach, because we expected a substantial cut-off point effect in the included studies. We did not want to be dependent upon the assumption that the diagnostic odds ratio's in our study would not be affected by differences in the individual cut-off points. To reduce heterogeneity we decided to choose one common cut-off point for all studies. We took a cut-off point of two standard deviations (SD) after consulting with nuclear imaging experts in our hospital and the University Hospital of Amsterdam. Both departments use a cut-off point of 2 SD's below healthy controls. So we recalculated all results from all studies using the individual data from tables and figures in the published paper, using this new cut-off point. If recalculation was not possible (when data for individual patients were not traceable from the manuscript), we excluded the study. This exclusion leads to bias, of course. We feel, however, that, as these studies did not adhere to recommended guidelines by not providing the raw scan results to allow the construction of the diagnostic 2 × 2 table, we did not exclude the methodologically best studies [24].

### Data extraction and analyses

Sensitivity, specificity and the odds ratio was calculated for each study separately, and the pooled odds ratio's (OR) for all studies together. Although we tried to reduce heterogeneity by recalculating study results using one common cut-off point, we still expected a threshold effect, because of differences in patients, SPECT machinery, radiotracers etc. Therefore, according to recommendations by Deekes and Egger we used diagnostic OR's [26].

**Figure 4:**
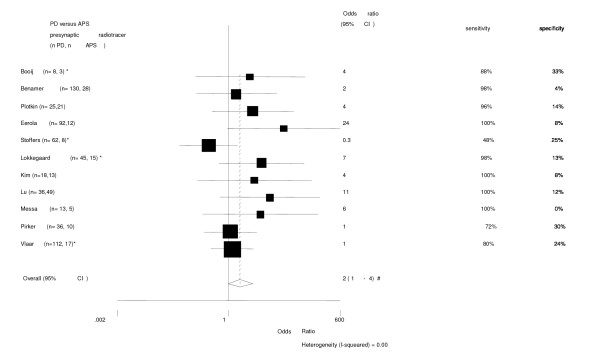
Diagnostic differentiation between PD and APS (MSA & PSP). Presynaptic radiotracer. [28, 30, 36, 38, 39, 46-48, 50-52]. * follow-up trial. # The pooled odds ratio after excluding the study of Messa '98 (sensitivity 100%, specificity 0%) remains unchanged.

**Figure 5:**
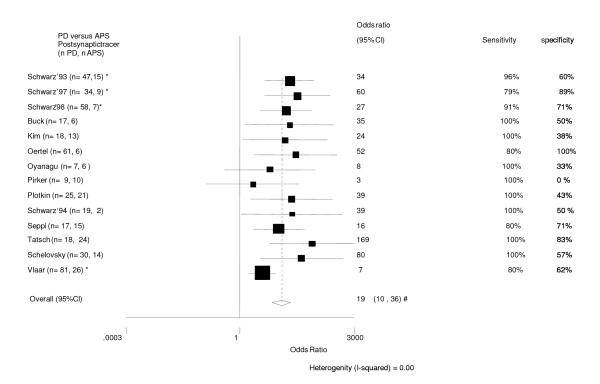
Diagnostic differentiation of patient with PD vs. APS (MSA & PSP). Postsynaptic radiotracers [18, 28, 34, 35, 37, 46, 50, 53-59]. * follow-up trial. # The pooled odds ratio (95%CI) after excluding the study of Pirker '97 (sensitivity 100%, specificity 0%) did not change significantly: 19 (10–33). Schwarz 1993. this trial can be seen as follow-up trial (long-term l-dopa is golden standard) but also as a cross-sectional trial. In the last case the results will be different (sensitivity = 79%, specificity = 100%). Schwarz 1997: We took signs (in) compatible with PD as golden standard. If taken long-term l-dopa as golden standard sensitivity is 100% and specificity is 67%. Schwarz 1998: We took signs (in) compatible with PD taken as golden standard, if taken long-term-l-dopa as golden standard sensitivity is 100% and specificity is 50%.

**Figure 6:**
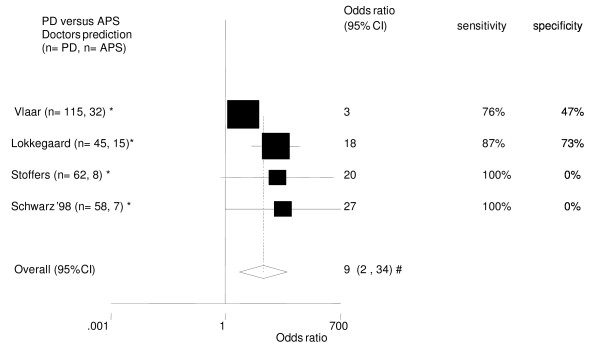
Diagnostic differentiation of patients with PD vs. APS (MSA & PSP). Doctors Prediction [28, 34, 38, 39]. * follow-up trial. # The pooled odds ratio (95%CI) after excluding the studies with a sensitivity of 100% and a specificity of 0% (Stoffers '05 and Schwarz '98) did not change significantly: 6 (1–40). Both trials diagnosed at the beginning of the follow-up all patients as having PD.

For studies with zeroes in one or more cells 0.5 was added to all four cells of the 2 × 2 table. Trials with a sensitivity of 100% and a specificity of 0% were not excluded, however the pooled OR's were also calculated without such studies (See # in Figure 4, 5 and 6).

All results were put in software SPSS 11.0 for Windows and later converted to Stata/SE9.

The metan and metareg commands were built in Stata/SE9. Because of the heterogeneity of the selected studies we used a random model to calculate the diagnostic odds ratios. Heterogeneity was calculated with the I ^2 ^[27].

## Results

### Literature search

The search on Medline (SPECT & clinical trial) gave 1503 hits. When we added all parkinsonian disorders we limited the Medline search to 56 relevant hits.

In the Cochrane database we found 26 hits and in Embase 45 hits, but no additional clinical studies above the ones found in Medline. With cross-reference searching we found an additional 128 relevant trials (See additional file 1). Together with our own retrospective study of 248 patients with unclear parkinsonism who underwent SPECT in the period 2001 to 2006, this resulted in 185 possibly relevant studies [28].

Of these 185 we excluded 153 studies (See additional file 2). Seven were excluded because of the language criteria and 85 articles were excluded as they did not deal with one of our three designated areas of clinical relevancy: most of them were about techniques, dementia or drug efficacy. We excluded an additional 61, because the absolute numbers with a cut-off point of 2 standard deviations below the control group were not available or could not be derived. We mailed the authors of the four studies with more than 85 patients, to ask for missing data [29-32], and received a response from 1 [30]. We wanted to acquire raw data from relatively large studies that would have a substantial impact on our meta-analysis; there were 4 large studies with more than 85 patients, the rest involved smaller numbers in the 20–35 range. We thus sought to contact the authors of these 4 studies, as we felt that studies with less than 85 subjects would have a very limited impact on the overall scores.

Of the 32 trials left (See additional file 3) 7 dealt with diagnostic uncertainty including a clinical follow-up as surrogate gold standard, 20 studies were cross-sectional including subjects with known parkinsonian disorders, and 6 studies involved patients with early PD. One clinical study fitted as well in the early PD groups as in the study group of known parkinsonian disorders [33]. Of the 7 follow-up studies, 5 were prospective. Of these prospective analyses, 2 included untreated new patients with parkinsonism [34,35], and 3 included patients with inconclusive parkinsonism or with a questionable effect of anti-parkinsonian medication [28,36,37]. Two of the 7 follow-up studies were retrospective: Lokkegaard and colleagues retrospectively investigated 90 consecutive patients referred for Beta-CIT SPECT for various reasons, and a non-treating neurologist obtained the final diagnosis from the clinical records of the patients [38]. Stoffers et al retrospectively analysed the SPECT scans of 72 patients with an initial diagnosis of PD, who were then re-diagnosed after various intervals [39]. The demographic and methodological characteristics of all included studies are visible in Table 1. The absolute numbers of the 2 × 2 tables of all included trials are shown in Table 2.

**Table 2:** Two by two tables for all included trials

	True positive	False negative	True negative	False positive
	
**Early PD vs normalcy**				
**Presynaptic**				
Asenbaum	23	6	30	0
Haapanimie	16	13	21	0
Huang	32	2	17	0
Muller	14	10	15	0
Schwarz '00	28	0	9	0
V. Laere (tracer FP-CIT)	15	24	10	0
V. Laere (tracer: TRODAT)	3	34	10	0
**PD vs ET**				
**Presynaptic**				
Booij '00	7	1	5	0
Benamer '00	127	3	25	2
Plotkin '05	24	1	11	0
Eerola '05	92	0	16	0
Vlaar '06	90	22	20	1
Lokkegaard '02	44	1	8	0
Asenbaum '98	23	6	32	0
Lee '99	10	1	12	3
**Postsynaptic**				
Vlaar '06	48	33	6	7
Plotkin '05	25	0	0	11
**PD vs VP**				
**Presynaptic**				
Vlaar '06	90	22	14	0
Booij '00	7	1	4	0
Eerola '05	92	0	11	4
Lokkegaard '02	44	1	3	1
Gerschlager '02	18	2	12	1
**Postsynaptic**				
Vlaar '06	65	16	8	4
**PD vs APS**				
**Presynaptic**				
Booij '00	7	1	1	2
Benamer '00	127	3	1	27
Plotkin '05	24	1	3	18
Eerola '05	92	0	1	11
Stoffers '05	30	32	2	6
Lokkegaard '02	44	1	2	13
Kim '02	18	0	1	12
Lu '04	36	0	6	43
Messa '98	13	0	0	5
Pirker '02	26	10	3	7
Vlaar '06	90	22	4	13
**postsynaptic**				
Schwarz '93	45	2	9	6
Schwarz '97	30	4	8	1
Schwarz '98	53	5	5	2
Buck '95	17	0	3	3
Kim '02	18	0	5	8
Oertel '93	49	12	6	0
Oyanagu '02	7	0	2	4
Pirker '97	9	0	0	10
Plotkin '05	25	0	9	12
Schwarz '94	19	0	1	1
Seppi '04	12	5	13	2
Tatsch '91	18	0	20	4
Schelovsky '93	30	0	8	6
Vlaar '06	65	16	16	10
**MSA vs PSP**				
**Presynaptic**				
Vlaar '06	19	0	1	9
Plotkin '05	7	0	1	5
Pirker	7	2	2	4
Pirker	18	0	1	9
Kim '02	11	2	1	7
Benamer '00	4	1	0	2
**Postsynaptic**				
Buck '95	5	2	6	0
Kim '02	1	1	2	2
Plotkin '05	7	6	6	2
Vlaar'06	10	4	3	6
v. Royen '93	13	4	1	3

* In the statistic analyses +0.5 was taken for the zeroes in the 2 × 2 table.

### Results of the pooled study data analysis

We recalculated the diagnostic power of SPECT for the following clinical problems: 1. diagnosis of PD in an early phase vs. normalcy; 2 diagnostic differentiation between PD and ET; 3. diagnostic differentiation between PD and vascular parkinsonism; 4. diagnostic differentiation between PD and Atypical Parkinsonian Syndromes (APS) consisting of MSA and PSP.

#### 1. Diagnosis of PD in an early phase vs. normalcy

**Figure 1:**
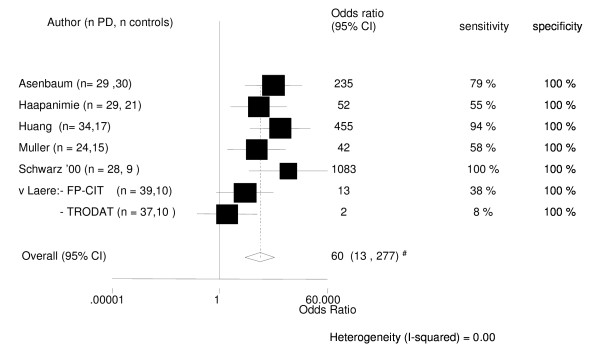
Diagnostic differentiation of patients with PD in an early phase vs. normalcy. Presynaptic radiotracer [33, 40-44]. # all studies have a specificity of 100% so the pooled odds ratio should therefore be infinite. The odds ratio of 60 is caused by STATA software's procedure for handling zero cells in the 2 × 2 table. abnormal SPECT definition: > 2 standard deviations below the bindingrate of healthy controls. outcome: sensitivity, specificity, odds ratio 95% confidence interval. surface square is based on the weight of the study. n = numbers of subjects. FP-CIT: Iodine-123-Ioflupane. TRODAT: 99mCT-TRODAT-1.

Pooled data from selected studies [33,40-44] were used to calculate the diagnostic accuracy of SPECT to differentiate between PD and normalcy. All six cross-sectionals (utilising presynaptic tracers) with patients with known PD in an early stage (Hoehn & Yahr score of 2 or lesser) had a specificity of 100%. However sensitivity varied from 8% to 100%. The pooled odds ratio with a 95%CI was 60 (13 – 277). See also Figure 1.

#### 2. Diagnostic differentiation between PD and essential tremor (ET)

Data from selected studies [33,36,38,45-48] and our own clinical follow-up study [28] were pooled and pooled odds ratios for diagnostic power of SPECT were calculated as described.

**Figure 2:**
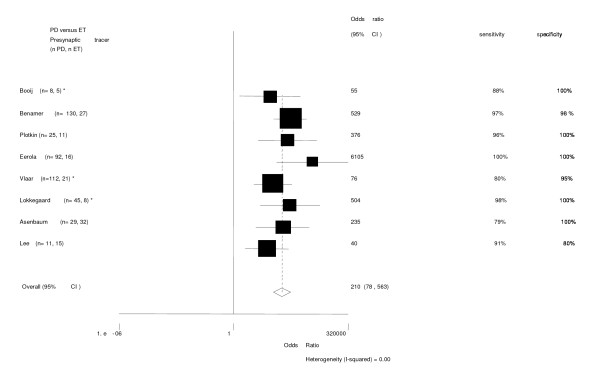
Patients with PD vs ET. Presynaptic radiotracer [36, 38, 45-48, 84]. * = follow-up trial.

For presynaptic radiotracers the pooled odds ratio with 95%CI of the 8 studies together was 210 (79–563). See also figure 2. All studies scored high sensitivity and specificity.

For calculation of these odds ratios in studies with postsynaptic radiotracers we found 2 studies: one cross-sectional study (sensitivity 100%, specificity of 0%) and our own follow-up study (sensitivity 60%, specificity 46%). The pooled odds ratio with 95%CI was 2 (0.4–5). [28,46]

Two studies compared the diagnostic accuracy of the treating physician with the SPECT in its capacity to delineate PD from ET [28,38]. Clinical diagnosis in these trials reached a sensitivity of respectively 76% and 87% and a specificity of 50% and 80%. The odds ratio with 95%CI of the two studies together is 9 (3–28).

#### 3. Diagnostic differentiation between PD and vascular parkinsonism (VP)

Pooled data from selected studies were used to calculate the diagnostic accuracy of SPECT to differentiate between PD and vascular parkinsonism (VP) [28,36,38,47,49].

**Figure 3:**
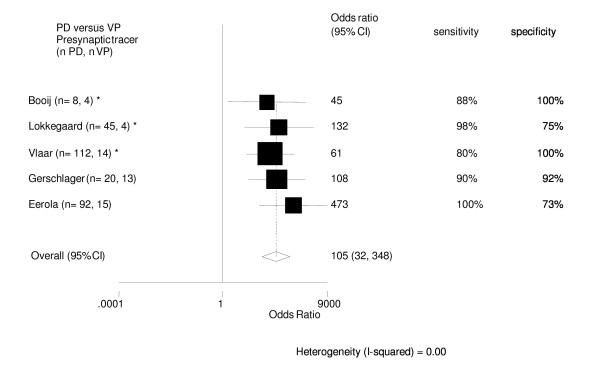
Diagnostic differentiation of patients with PD versus VP. Presynaptic tracer. [28, 36, 38, 47, 49]. * = follow-up trial.

Using presynaptic radiotracers the 5 studies all scored high sensitivity and specificity for SPECT to differentiate between PD and VP. The pooled odds ratio with 95%CI of all five studies together is 105 (32 – 348). See also figure 3.

We were not able to find any trials except our own trial with postsynaptic tracers aimed at this diagnostic problem. We found a sensitivity of 80%, specificity of 67% and an odds ratio of 95%CI of 8 (2 – 30).

Lokkegaard et al. and we mentioned the diagnostic accuracy of the clinician to differentiate PD from VP and found a sensitivity of respectively 87% and 76% and a specificity of respectively 0% and 63% with a odds ratio 95%CI of 3 (0.5–18) [28,38].

#### 4. Diagnostic differentiation between PD and APS (MSA & PSP)

Pooled data from selected studies were used to calculate the diagnostic accuracy of SPECT to differentiate between PD and APS [18,28,30,34-39,46-48,50-59].

When using presynaptic tracers all trials scored moderate to high sensitivity, but with a low specificity. Combining all 11 trials the mean odds ratio with 95% CI for the presynaptic tracer to differentiate between PD and the two APS was 2 (1 – 4). See Figure 4.

When using postsynaptic tracers the 14 trial scored together a pooled odds ratio 95% CI of 19 (9–36). See Figure 5.

Four follow-up studies compared the diagnostic accuracy of the treating physician with the SPECT in its capacity to delineate PD from the two APS. Clinical diagnosis in these trials reached a high sensitivity, but a low specificity. The pooled odds ratio 95% CI was 9 (2 – 34). See Figure 6.

The diagnostic accuracy of SPECT to differentiate MSA from PSP was low for both the presynaptic and postsynaptic radiotracers. With presynaptic racers sensitivity of the 6 trials was extremely high (78 – 100%), however specificity was low (0–33%). The 5 studies with postsynaptic tracers scored moderate sensitivity (50 – 71%) and specificity (25 – 100%).

The pooled odds ratio with 95% CI was 2 (0.6 – 7) for the presynaptic tracers and 2 (0.8 – 6) for the postsynaptic tracers [28,46,48,50,52,56,60,61].

## Discussion

To our knowledge this is the first meta-analysis of the literature on the diagnostic value of SPECT in patients with parkinsonian syndromes. Before discussing the actual results we would like to point out some methodological problems.

We were very strict in including studies. To compare the results from selected studies we defined positive tests as values equal with or exceeding two standard deviations below healthy controls. When authors defined their positive results otherwise, we recalculated these, where possible. However, as many studies did not include raw data, we had to reject these, as we were then not able to (re)calculate the absolute numbers of true positive, false negative, true negative, and false positives with a cut-off point of two standard deviations.

We dichotomised the radiotracers utilised into two groups: presynaptic and postsynaptic tracers. By doing this we assumed that all tracers in one group have a similar reliability. This seems to be reasonable for beta-CIT and FP-CIT, especially when the SPECT of the parkinsonian patient is expressed as a percentage of the binding ratios found in healthy controls [62,63].

Besides the use of different equipment, comparison between studies is also hampered by the different methods that investigators use to calculate the tracer binding. In some studies the striatum was encircled manually and others used predefined templates. The striatum was judged visually (compatible or not compatible with PD) or fully automated quantitatively by others. Finally, and possibly the greatest confounding variable: in some studies the SPECT results are judged by investigators unaware of the clinical records, but in more than half of the studies blinding of the investigator is not mentioned.

Another methodological shortcoming in our meta-analysis is the great variability in clinical criteria used to classify patients; many authors do not even mention these. A major issue here is that most studies use clearly-defined later-stage patients that are obviously not representative for the diagnostic problem that one wants to solve with a SPECT. We thus limited our analysis to those studies that addressed the diagnostic accuracy in clinically relevant situations: early PD, follow-up studies and studies with known parkinsonian diseases. All the measures mentioned above were taken to minimise heterogeneity, which was actually shown to be minimal as calculated by the I^2 ^statistic [27]. Despite this, interpreting of the results should be done with caution, as postsynaptic tracer binding in APS can be normal in an early stage of disease with a decrease later on [64]. Finally, strict age-matching is not done in all studies, but is mandatory as tracer binding in general decreases with age [65-67].

These methodological problems do, however, allow one important conclusion to start with: as we derived only 32 papers suitable from a starting number of 185, there is a paucity of methodologically sound and clinically relevant papers on this subject. Below we would like to discuss our results according to the predefined clinically relevant situations.

### Diagnosis of PD in an early phase vs. normalcy

Our meta-analysis confirms the general opinion that SPECT is relatively accurate to differentiate between patients with PD in an early stage and healthy controls. The difference in sensitivity between trials can not be explained solely by different radiotracer usage. Especially the difference with the two studies using TRODAT is obvious [41,43]. An explanation may be that the use of a template vs. hand-circling of the striatum leads to lower diagnostic specificities [40,42,43]. Other possible explanations for the lower sensitivity scores in the study of van Laere et al are their consecutive inclusion of patients and their clinic being a tertiary referral centre [43].

Schwarz and Asenbaum were the only two authors who used two standard deviations below the normal controls as cut-off [33,42]. In the other four studies we recalculated the absolute numbers of true positive, false negative etc. by ourselves, [40,41,43,44] which led in all 4 studies to lower numbers for diagnostic accuracy. Apart from this different cut-off point, higher sensitivity figures in several large trials (normal SPECT scans in 5–10% of clinically definitive PD patients) are probably explained by the disease stage of the patients [68-70].

Asenbaum's, Haapaniemi's and Muller's were the only studies which mentioned blinding of the investigators [33,40,44]. It is perhaps not surprising that these authors found lower numbers for diagnostic accuracy than Huang and Schwarz (See fig. 1) [41,42].

### Diagnosis of PD versus ET

The results of our meta-analysis confirm the general opinion that SPECT with presynaptic tracers is highly accurate to differentiate between patients with PD and ET. Lee et al. scored lowest specificity. A possible explanation is that they included not only patients with ET but as well patients with isolated postural tremor and postural in combination with resting tremor [45].

### Diagnostic differentiation between patients with PD versus VP

According to the results of the meta-analysis we conclude that presynaptic SPECT scans can accurately differentiate between patients with PD and VP. The specificity, however, is only moderate in the studies of Lokkegaard and Eerola [38,47]. VP is a somewhat controversial clinical concept and the differences found in the studies we analysed probably reflect the variability in clinical definition. Whereas Lokkegaard et al., Gerschlager et al., and Eerola et al. [38,49] used strict inclusion criteria, Booij et al. did not [36,47]. This point is illustrated in the paper by Loberboym et al, who investigated 20 patients with VP with FP-CIT SPECT. Nine had a normal presynaptic SPECT scan but 11 had decreased striatal tracer binding. All nine patients with normal presynaptic SPECT scan had no reaction on levodopa treatment, however 5 of the 11 with decreased striatal FP-CIT binding did have [71]. Although SPECT with presynaptic tracers scored high accuracy in differentiation between PD and VP, conventional techniques like CT and MRI may still be necessary as additional diagnostic tools.

### Diagnostic differentiation between patients with PD versus APS

This meta-analysis confirmed the generally accepted view that presynaptic tracers cannot distinguish between PD and APS. However, we also found that postsynaptic SPECT is not very good at this. A negative postsynaptic SPECT scan does not exclude APS. The positive predictive value of abnormal postsynaptic SPECT for the diagnosis of APS is high, making a reduced postsynaptic radiotracer binding likely to exclude a diagnosis of PD. However, patients with PD in our meta-analysis do show loss of dopamine-receptor binding. Studies which used IBZM scored higher accuracy than studies with IBF or Epidepride [50,53,56,59]. If the trials are excluded which used other tracers than IBZM the mean 95%CI odds ratio only increased to 21 (11–44).

Some studies, excluded for this meta-analysis, found excellent accuracy for the postsynaptic tracer to differentiate between PD and APS [19,64,72-77]. Schulz et al, who investigated 32 MSA patients, found similar results as in our meta-analysis: only a significant loss in 63% of the patients using IBZM-SPECT with two standard deviations under controls (PD patients) as cut-off point [78]. Results of the studies by Berding et al [79], and Bettin et al [80], are in line with these.

Possible explanations for the difference in results may be difference in cut-off points (many authors use only one SD below healthy controls), and the use of special techniques claimed by some to enhance diagnostic accuracy, e.g. asymmetric indices, caudate/putamen atio, presynaptic/postsynaptic ratio of tracer binding, speed of decline in follow-up [30,36,76,81,82]. One other explanation of the low discriminating value of postsynaptic SPECT imaging is the reversible down regulation of dopamine receptors by dopaminergic drugs [83]. If the drugs are not stopped appropriately, the scan result can be false positive. In our meta-analysis we found 9 studies where potentially interfering medication was not discontinued appropriately or these did even not mention whether this medication was discontinued (and if, for how long) before the scan.

## Conclusion

There is a paucity of methodologically sound and clinically relevant papers on the diagnostic accuracy of SPECT in parkinsonian syndromes. Our meta-analysis confirms the general opinion that SPECT is relatively accurate to differentiate between patients with PD in an early stage and healthy controls. We were also able to confirm the general opinion that SPECT with presynaptic tracers is highly accurate to differentiate between patients with PD and ET. Although SPECT with presynaptic tracers scored high accuracy in differentiation between PD and VP, conventional techniques like CT and MRI may still be necessary as additional diagnostic tools. This meta-analysis confirmed the generally accepted view that presynaptic tracers cannot distinguish between PD and APS. However, we also found that SPECT with postsynaptic tracers is not very good at this.

## Abbreviations

Beta-CIT 123I-Iodine-beta-CIT (presynaptic radiotracer)

CI confidence interval

CT computer tomography

DIP drug induced parkinsonism

ET essential tremor

FP-CIT 123I-ioflupane (presynaptic radiotracer)

IBF 123I-idiobenzofuran (postsynaptic radiotracer)

IBZM 123I-iodobenzamide (postsynaptic radiotracer)

PD idiopathic Parkinson disease

MRI magnetic resonance imaging

MSA multiple system atrophy

NPV negative predictive value

OR odds ratio

PET positron emission tomography

PPV positive predictive value

PSP progressive supranuclear palsy

SD standard deviation

SPECT single photon emission computer tomography

TRODAT 99mCT-TRODAT-1 (presynaptic radiotracer)

VP vascular parkinsonism

## Competing interests

The author(s) declare that they have no competing interests.

## Authors' contributions

WW and MvK initiated the study. WW, AV and AK wrote the protocol, AV did the literature search and paper retrieval, AV and WW judged each paper, AV and AK did the statistical calculations. AV wrote a first draft of the paper, which was finished in its final form by WW.

All authors have read and approved the manuscript.

## Pre-publication history

The pre-publication history for this paper can be accessed here:



## Supplementary Material

Additional file 1 flowchart. flowchart of the study selection for the meta-analysis. Click here for file

Additional file 2 reference list 1. reference list of the studies excluded for the meta-analysis Click here for file

Additional file 3 reference list 2. reference list of the studies included in the meta-analysis Click here for file
