# Supplementary material for: Meta-analysis of the literature on diagnostic accuracy of SPECT in parkinsonian syndromes
Source: BMC Neurol. 2007 Sep 1;7:27. doi: 10.1186/1471-2377-7-27 (PMC2064928; doi:10.1186/1471-2377-7-27)
Supplement: Additional file 1 — flowchart. flowchart of the study selection for the meta-analysis. [file 1471-2377-7-27-S1.doc]

Flowchart 1

Result of literature searching

Embase

45 hits,

0 new hits

Cochrane

26 hits,

0 new hits

N= 8

PD vs ET

language

n=7

none of the 3 subgroups

n= 85

153 of the 185 were excluded because:

N= 5

PD vs VP

32 studies included:

6 early PD

7 follow-up

20 known parkinsonian

(1 used in 2 groups)

N= 9

PD, MSA & PSP versus other

N= 6

Patients with early PD

Reference searching

128 new hits

Own retrospective trial

Pubmed

56 hits

no absolute numbers

n= 61

N=22

PD vs MSA & PSP
