# Supplementary material for: Meta-analysis of the literature on diagnostic accuracy of SPECT in parkinsonian syndromes
Source: BMC Neurol. 2007 Sep 1;7:27. doi: 10.1186/1471-2377-7-27 (PMC2064928; doi:10.1186/1471-2377-7-27)
Supplement: Additional file 2 — reference list 1. reference list of the studies excluded for the meta-analysis [file 1471-2377-7-27-S2.doc]

Excluded trials

[1-152]

1. **Pramipexole vs levodopa as initial treatment for Parkinson disease: A randomized controlled trial. Parkinson Study Group**. *Jama* 2000, **284**:1931-8.

2. **A randomized controlled trial comparing pramipexole with levodopa in early Parkinson's disease: design and methods of the CALM-PD Study. Parkinson Study Group**. *Clin Neuropharmacol* 2000, **23**:34-44.

3. **Dopamine transporter brain imaging to assess the effects of pramipexole vs levodopa on Parkinson disease progression**. *Jama* 2002, **287**:1653-61.

4. A Abi-Dargham, RB Innis, G Wisniewski, RM Baldwin, JL Neumeyer, JP Seibyl: **Human biodistribution and dosimetry of iodine-123-fluoroalkyl analogs of beta-CIT**. *Eur J Nucl Med* 1997, **24**:1422-5.

5. JE Ahlskog, DM Maraganore, RJ Uitti, GR Uhl: **Brain imaging to assess the effects of dopamine agonists on progression of Parkinson disease**. *Jama* 2002, **288**:311; author reply 312-3.

6. JE Ahlskog, RJ Uitti, MK O'Connor, DM Maraganore, JY Matsumoto, KF Stark, MF Turk, OL Burnett: **The effect of dopamine agonist therapy on dopamine transporter imaging in Parkinson's disease**. *Mov Disord* 1999, **14**:940-6.

7. A Antonini, R Benti, R De Notaris, S Tesei, A Zecchinelli, G Sacilotto, N Meucci, M Canesi, C Mariani, G Pezzoli, et al: **123I-Ioflupane/SPECT binding to striatal dopamine transporter (DAT) uptake in patients with Parkinson's disease, multiple system atrophy, and progressive supranuclear palsy**. *Neurol Sci* 2003, **24**:149-50.

8. A Antonini, A Landi, R Benti, C Mariani, R De Notaris, G Marotta, G Pezzoli, SM Gaini, P Gerundini: **Functional neuroimaging (PET and SPECT) in the selection and assessment of patients with Parkinson's disease undergoing deep brain stimulation**. *J Neurosurg Sci* 2003, **47**:40-6.

9. G Arnold, K Tatsch, WH Oertel, T Vogl, J Schwarz, E Kraft, CM Kirsch: **Clinical progressive supranuclear palsy: differential diagnosis by IBZM-SPECT and MRI**. *J Neural Transm Suppl* 1994, **42**:111-8.

10. S Asenbaum, T Brucke, W Pirker, I Podreka, P Angelberger, S Wenger, C Wober, C Muller, L Deecke: **Imaging of dopamine transporters with iodine-123-beta-CIT and SPECT in Parkinson's disease**. *J Nucl Med* 1997, **38**:1-6.

11. H Barthel, U Muller, T Wachter, P Slomka, C Dannenberg, T Murai, T Kahn, P Georgi: **[Multimodal SPECT and MRT imaging data analysis for an improvement in the diagnosis of idiopathic Parkinson's syndrome]**. *Radiologe* 2000, **40**:863-9.

12. JL Baulieu, MJ Ribeiro, C Levilion-Prunier, F Tranquart, JR Chartier, D Guilloteau, JP Cottier, JC Besnard, L Pourcelot, A Autret: **Effects of the method of drawing regions of interest on the differential diagnosis of extrapyramidal syndromes using 123I-iodolisuride SPET**. *Nucl Med Commun* 1999, **20**:77-84.

13. HT Benamer, WH Oertel, J Patterson, DM Hadley, O Pogarell, H Hoffken, A Gerstner, DG Grosset: **Prospective study of presynaptic dopaminergic imaging in patients with mild parkinsonism and tremor disorders: part 1. Baseline and 3-month observations**. *Mov Disord* 2003, **18**:977-84.

14. HT Benamer, J Patterson, DJ Wyper, DM Hadley, GJ Macphee, DG Grosset: **Correlation of Parkinson's disease severity and duration with 123I-FP-CIT SPECT striatal uptake**. *Mov Disord* 2000, **15**:692-8.

15. G Berding, T Brucke, P Odin, DJ Brooks, H Kolbe, P Gielow, H Harke, BO Knoop, R Dengler, WH Knapp: **[[123I]beta-CIT SPECT imaging of dopamine and serotonin transporters in Parkinson's disease and multiple system atrophy**. *Nuklearmedizin* 2003, **42**:31-8.

16. G Berding, KF Gratz, H Kolbe, GJ Meyer, R Dengler, BO Knoop, H Hundeshagen: **123I-IBZM SPECT: reconstruction methodology and results in parkinsonism and dystonia**. *Nuklearmedizin* 1994, **33**:194-9.

17. HW Berendse, J Booij, CM Francot, PL Bergmans, R Hijman, JC Stoof, EC Wolters: **Subclinical dopaminergic dysfunction in asymptomatic Parkinson's disease patients' relatives with a decreased sense of smell**. *Ann Neurol* 2001, **50**:34-41.

18. S Bettin, I Kampfer, A Seese, A Schafer, M Reuter, J Lossner, J Dietrich, A Wagner, WH Knapp: **[Striatal uptake of I-123-beta-CIT and I-123-IBZM in patients with extrapyramidal symptoms]**. *Nuklearmedizin* 1997, **36**:167-72.

19. B Bokobza, M Ruberg, B Scatton, F Javoy-Agid, Y Agid: **[3H]spiperone binding, dopamine and HVA concentrations in Parkinson's disease and supranuclear palsy**. *Eur J Pharmacol* 1984, **99**:167-75.

20. J Booij, G Tissingh, GJ Boer, JD Speelman, JC Stoof, AG Janssen, EC Wolters, EA van Royen: **[123I]FP-CIT SPECT shows a pronounced decline of striatal dopamine transporter labelling in early and advanced Parkinson's disease**. *J Neurol Neurosurg Psychiatry* 1997, **62**:133-40.

21. J Booij, G Tissingh, A Winogrodzka, GJ Boer, JC Stoof, EC Wolters, EA van Royen: **Practical benefit of [123I]FP-CIT SPET in the demonstration of the dopaminergic deficit in Parkinson's disease**. *Eur J Nucl Med* 1997, **24**:68-71.

22. T Bosman, K Van Laere, P Santens: **Anatomically standardised 99mTc-ECD brain perfusion SPET allows accurate differentiation between healthy volunteers, multiple system atrophy and idiopathic Parkinson's disease**. *Eur J Nucl Med Mol Imaging* 2003, **30**:16-24.

23. T Brucke, S Asenbaum, W Pirker, S Djamshidian, S Wenger, C Wober, C Muller, I Podreka: **Measurement of the dopaminergic degeneration in Parkinson's disease with [123I] beta-CIT and SPECT. Correlation with clinical findings and comparison with multiple system atrophy and progressive supranuclear palsy**. *J Neural Transm Suppl* 1997, **50**:9-24.

24. T Brucke, S Djamshidian, G Bencsits, W Pirker, S Asenbaum, I Podreka: **SPECT and PET imaging of the dopaminergic system in Parkinson's disease**. *J Neurol* 2000, **247 Suppl 4**:IV/2-7.

25. T Brucke, I Podreka, P Angelberger, S Wenger, A Topitz, B Kufferle, C Muller, L Deecke: **Dopamine D2 receptor imaging with SPECT: studies in different neuropsychiatric disorders**. *J Cereb Blood Flow Metab* 1991, **11**:220-8.

26. AM Catafau, E Tolosa: **Impact of dopamine transporter SPECT using 123I-Ioflupane on diagnosis and management of patients with clinically uncertain Parkinsonian syndromes**. *Mov Disord* 2004, **19**:1175-82.

27. R Ceravolo, D Volterrani, G Gambaccini, S Bernardini, C Rossi, C Logi, G Tognoni, G Manca, G Mariani, U Bonuccelli, et al: **Presynaptic nigro-striatal function in a group of Alzheimer's disease patients with parkinsonism: evidence from a dopamine transporter imaging study**. *J Neural Transm* 2004, **111**:1065-73.

28. KL Chou, HI Hurtig, MB Stern, A Colcher, B Ravina, A Newberg, PD Mozley, A Siderowf: **Diagnostic accuracy of [99mTc]TRODAT-1 SPECT imaging in early Parkinson's disease**. *Parkinsonism Relat Disord* 2004, **10**:375-9.

29. M Chouker, K Tatsch, R Linke, O Pogarell, K Hahn, J Schwarz: **Striatal dopamine transporter binding in early to moderately advanced Parkinson's disease: monitoring of disease progression over 2 years**. *Nucl Med Commun* 2001, **22**:721-5.

30. SJ Colloby, JT O'Brien, JD Fenwick, MJ Firbank, DJ Burn, IG McKeith, ED Williams: **The application of statistical parametric mapping to 123I-FP-CIT SPECT in dementia with Lewy bodies, Alzheimer's disease and Parkinson's disease**. *Neuroimage* 2004, **23**:956-66.

31. M Contin, P Martinelli, M Mochi, F Albani, R Riva, C Scaglione, M Dondi, S Fanti, C Pettinato, A Baruzzi: **Dopamine transporter gene polymorphism, spect imaging, and levodopa response in patients with Parkinson disease**. *Clin Neuropharmacol* 2004, **27**:111-5.

32. M Contin, P Martinelli, R Riva, M Dondi, S Fanti, C Pettinato, C Scaglione, F Albani, A Baruzzi: **Assessing dopaminergic function in Parkinson's disease: levodopa kinetic-dynamic modeling and SPECT**. *J Neurol* 2003, **250**:1475-81.

33. M Cordes, H Henkes, D Laudahn, H Brau, W Kramp, W Girke, J Hierholzer, H Eichstadt, R Felix: **Initial experience with SPECT examinations using [123I]IBZM as a D2-dopamine receptor antagonist in Parkinson's disease**. *Eur J Radiol* 1991, **12**:182-6.

34. M Cordes, J Hierholzer, L Schelosky, W Poewe, I Cordes, R Horowski, H Eichstaedt, D Schmidt, R Felix: **IBZM-SPECT imaging in Parkinson's disease. Quantification of binding ratios from sequential SPECT measurements in patients and controls**. *Adv Neurol* 1993, **60**:525-8.

35. M Cordes, J Hierholzer, L Schelosky, A Schrag, WS Richter, H Eichstadt, PE Schulze, W Poewe, R Felix: **Iodine-123-iodo-lisuride SPECT in Parkinson's disease**. *J Nucl Med* 1996, **37**:22-5.

36. A Druschky, MJ Hilz, G Platsch, M Radespiel-Troger, K Druschky, T Kuwert, B Neundorfer: **Differentiation of Parkinson's disease and multiple system atrophy in early disease stages by means of I-123-MIBG-SPECT**. *J Neurol Sci* 2000, **175**:3-12.

37. K Easterford, P Clough, M Kellett, K Fallon, S Duncan: **Reversible parkinsonism with normal beta-CIT-SPECT in patients exposed to sodium valproate**. *Neurology* 2004, **62**:1435-7.

38. I Eisensehr, R Linke, S Noachtar, J Schwarz, FJ Gildehaus, K Tatsch: **Reduced striatal dopamine transporters in idiopathic rapid eye movement sleep behaviour disorder. Comparison with Parkinson's disease and controls**. *Brain* 2000, **123 ( Pt 6)**:1155-60.

39. EG Eising, TH Muller, L Freudenberg, SP Muller, K Dutschka, W Sonnenschein, H Przuntek, A Bockisch: **SPECT imaging with [123I]-beta-CIT in Parkinsonism: comparison of SPECT images obtained by a single-headed and a three-headed gamma camera**. *Nucl Med Commun* 2001, **22**:145-50.

40. EG Eising, TT Muller, C Zander, W Kuhn, J Farahati, C Reiners, HH Coenen: **SPECT-evaluation of the monoamine uptake site ligand [123I](1R)-2-beta-carbomethoxy-3-beta-(4-iodophenyl)-tropane ([123I]beta-CIT) in untreated patients with suspicion of Parkinson disease**. *J Investig Med* 1997, **45**:448-52.

41. S Fahn, D Oakes, I Shoulson, K Kieburtz, A Rudolph, A Lang, CW Olanow, C Tanner, K Marek: **Levodopa and the progression of Parkinson's disease**. *N Engl J Med* 2004, **351**:2498-508.

42. PA Fall, S Ekberg, AK Granerus, G Granerus: **ECT in Parkinson's disease-dopamine transporter visualised by [123I]-beta-CIT SPECT**. *J Neural Transm* 2000, **107**:997-1008.

43. A Feigin, A Antonini, M Fukuda, R De Notaris, R Benti, G Pezzoli, MJ Mentis, JR Moeller, D Eidelberg: **Tc-99m ethylene cysteinate dimer SPECT in the differential diagnosis of parkinsonism**. *Mov Disord* 2002, **17**:1265-70.

44. AJ Fischman, AA Bonab, JW Babich, EP Palmer, NM Alpert, DR Elmaleh, RJ Callahan, SA Barrow, W Graham, PC Meltzer, et al: **Rapid detection of Parkinson's disease by SPECT with altropane: a selective ligand for dopamine transporters**. *Synapse* 1998, **29**:128-41.

45. KA Frey, RA Koeppe, MR Kilbourn, TM Vander Borght, RL Albin, S Gilman, DE Kuhl: **Presynaptic monoaminergic vesicles in Parkinson's disease and normal aging**. *Ann Neurol* 1996, **40**:873-84.

46. AM Garcia Vicente, J Vaamonde Cano, VM Poblete Garcia, S Rodado Marina, M Cortes Romera, S Ruiz Solis, R Ibanez Alonso, A Soriano Castrejon: **[Utility of dopamine transporter imaging (123-I Ioflupane SPECT) in the assessment of movement disorders]**. *Rev Esp Med Nucl* 2004, **23**:245-52.

47. Y Geng, GH Shi, Y Jiang, LX Xu, XY Hu, YQ Shao: **Investigating the role of 99mTc-TRODAT-1 SPECT imaging in idiopathic Parkinson's disease**. *J Zhejiang Univ Sci B* 2005, **6**:22-7.

48. D Giobbe, GC Castellano, V Podio: **Dopamine D2 receptor imaging with SPECT using IBZM in 16 patients with Parkinson disease**. *Ital J Neurol Sci* 1993, **14**:165-9.

49. M Guttman, D Stewart, D Hussey, A Wilson, S Houle, S Kish: **Influence of L-dopa and pramipexole on striatal dopamine transporter in early PD**. *Neurology* 2001, **56**:1559-64.

50. T Hamano, T Tsuchida, M Hirayama, J Fujiyama, T Mutoh, Y Yonekura, M Kuriyama: **[Dopamine transporter SPECT in patients with Parkinson's disease]**. *Kaku Igaku* 2000, **37**:125-9.

51. RA Hauser, WC Koller, JP Hubble, T Malapira, K Busenbark, CW Olanow: **Time course of loss of clinical benefit following withdrawal of levodopa/carbidopa and bromocriptine in early Parkinson' s disease**. *Mov Disord* 2000, **15**:485-9.

52. A Hertel, M Weppner, H Baas, M Schreiner, FD Maul, RP Baum, PA Fischer, G Hor: **Quantification of IBZM dopamine receptor SPET in de novo Parkinson patients before and during therapy**. *Nucl Med Commun* 1997, **18**:811-22.

53. J Hierholzer, L Castelli, M Cordes, L Schelosky, W Poewe, R Felix: **[Cerebral SPECT with iodine-123 IBZM in patients with extrapyramidal system disorders: the evaluation of its sensitivity in therapy with dopaminergic drugs]**. *Radiol Med (Torino)* 1996, **91**:207-10.

54. J Hierholzer, M Cordes, L Schelosky, G Barzen, W Poewe, H Henkes, U Keske, R Horowski, R Felix: **[The determination of cerebral dopamine (D2) receptor density by using 123I-IBZM-SPECT in Parkinson disease patients]**. *Rofo* 1992, **157**:390-8.

55. J Hierholzer, M Cordes, L Schelosky, W Richter, A Schrag, W Poewe, PE Schulze, W Semmler, H Eichstadt, R Felix: **[Brain SPECT with 123I-lisuride in patients with Parkinson's disease and controls]**. *Nuklearmedizin* 1995, **34**:141-5.

56. J Hierholzer, M Cordes, L Schelosky, B Sander, JC Bock, I David, R Horowski, W Poewe: **[The differential diagnosis of Parkinson diseases--123I-IBZM-SPECT vs. the apomorphine test]**. *Rofo* 1993, **159**:86-90.

57. J Hierholzer, M Cordes, S Venz, L Schelosky, C Harisch, W Richter, U Keske, N Hosten, J Maurer, W Poewe, et al: **Loss of dopamine-D2 receptor binding sites in Parkinsonian plus syndromes**. *J Nucl Med* 1998, **39**:954-60.

58. WS Huang, YH Chiang, JC Lin, YH Chou, CY Cheng, RS Liu: **Crossover study of (99m)Tc-TRODAT-1 SPECT and (18)F-FDOPA PET in Parkinson's disease patients**. *J Nucl Med* 2003, **44**:999-1005.

59. M Ichise, JR Ballinger: **SPECT imaging of dopamine receptors**. *J Nucl Med* 1996, **37**:1591-5.

60. M Ichise, JR Ballinger, D Vines, S Tsai, HF Kung: **Simplified quantification and reproducibility studies of dopamine D2-receptor binding with iodine-123-IBF SPECT in healthy subjects**. *J Nucl Med* 1997, **38**:31-7.

61. M Ichise, YJ Kim, JR Ballinger, D Vines, SS Erami, F Tanaka, AE Lang: **SPECT imaging of pre- and postsynaptic dopaminergic alterations in L-dopa-untreated PD**. *Neurology* 1999, **52**:1206-14.

62. M Ichise, YJ Kim, SS Erami, JR Ballinger, D Vines, F Tanaka, AE Lang: **Functional morphometry of the striatum in Parkinson's disease on three-dimensional surface display of 123I-beta-CIT SPECT data**. *J Nucl Med* 1999, **40**:530-8.

63. Y Imon, H Matsuda, M Ogawa, D Kogure, N Sunohara: **SPECT image analysis using statistical parametric mapping in patients with Parkinson's disease**. *J Nucl Med* 1999, **40**:1583-9.

64. R Innis, R Baldwin, E Sybirska, Y Zea, M Laruelle, M al-Tikriti, D Charney, S Zoghbi, E Smith, G Wisniewski, et al: **Single photon emission computed tomography imaging of monoamine reuptake sites in primate brain with [123I]CIT**. *Eur J Pharmacol* 1991, **200**:369-70.

65. RB Innis, KL Marek, K Sheff, S Zoghbi, J Castronuovo, A Feigin, JP Seibyl: **Effect of treatment with L-dopa/carbidopa or L-selegiline on striatal dopamine transporter SPECT imaging with [123I]beta-CIT**. *Mov Disord* 1999, **14**:436-42.

66. RB Innis, JP Seibyl, BE Scanley, M Laruelle, A Abi-Dargham, E Wallace, RM Baldwin, Y Zea-Ponce, S Zoghbi, S Wang, et al: **Single photon emission computed tomographic imaging demonstrates loss of striatal dopamine transporters in Parkinson disease**. *Proc Natl Acad Sci U S A* 1993, **90**:11965-9.

67. T Ishikawa, V Dhawan, K Kazumata, T Chaly, F Mandel, J Neumeyer, C Margouleff, B Babchyck, I Zanzi, D Eidelberg: **Comparative nigrostriatal dopaminergic imaging with iodine-123-beta CIT-FP/SPECT and fluorine-18-FDOPA/PET**. *J Nucl Med* 1996, **37**:1760-5.

68. J Jankovic, AH Rajput, MP McDermott, DP Perl: **The evolution of diagnosis in early Parkinson disease. Parkinson Study Group**. *Arch Neurol* 2000, **57**:369-72.

69. DL Jennings, JP Seibyl, D Oakes, S Eberly, J Murphy, K Marek: **(123I) beta-CIT and single-photon emission computed tomographic imaging vs clinical evaluation in Parkinsonian syndrome: unmasking an early diagnosis**. *Arch Neurol* 2004, **61**:1224-9.

70. H Kageyama, S Kikuchi, K Tashiro: **[Analysis of Parkinson's disease and related syndromes using 123I-IMP-SPECT with the ARG method]**. *Nippon Rinsho* 1997, **55**:238-42.

71. PF Kao, KY Tzen, TC Yen, CS Lu, YH Weng, SP Wey, G Ting: **The optimal imaging time for [99Tcm]TRODAT-1/SPET in normal subjects and patients with Parkinson's disease**. *Nucl Med Commun* 2001, **22**:151-4.

72. R Katzenschlager, D Costa, W Gerschlager, J O'Sullivan, J Zijlmans, S Gacinovic, W Pirker, A Wills, K Bhatia, AJ Lees, et al: **[123I]-FP-CIT-SPECT demonstrates dopaminergic deficit in orthostatic tremor**. *Ann Neurol* 2003, **53**:489-96.

73. LS Kegeles, Y Zea-Ponce, A Abi-Dargham, J Rodenhiser, T Wang, R Weiss, RL Van Heertum, JJ Mann, M Laruelle: **Stability of [123I]IBZM SPECT measurement of amphetamine-induced striatal dopamine release in humans**. *Synapse* 1999, **31**:302-8.

74. GM Kim, SE Kim, WY Lee: **Preclinical impairment of the striatal dopamine transporter system in sporadic olivopontocerebellar atrophy: studied with [(123)I]beta-CIT and SPECT**. *Eur Neurol* 2000, **43**:23-9.

75. HJ Kim, JH Im, SO Yang, DH Moon, JS Ryu, JK Bong, KP Nam, JH Cheon, MC Lee, HK Lee: **Imaging and quantitation of dopamine transporters with iodine-123-IPT in normal and Parkinson's disease subjects**. *J Nucl Med* 1997, **38**:1703-11.

76. SE Kim, JY Choi, YS Choe, Y Choi, WY Lee: **Serotonin transporters in the midbrain of Parkinson's disease patients: a study with 123I-beta-CIT SPECT**. *J Nucl Med* 2003, **44**:870-6.

77. SE Kim, WY Lee, YS Choe, JH Kim: **SPECT measurement of iodine-123-beta-CIT binding to dopamine and serotonin transporters in Parkinson's disease: correlation with symptom severity**. *Neurol Res* 1999, **21**:255-61.

78. MB Knable, DW Jones, R Coppola, TM Hyde, KS Lee, J Gorey, DR Weinberger: **Lateralized differences in iodine-123-IBZM uptake in the basal ganglia in asymmetric Parkinson's disease**. *J Nucl Med* 1995, **36**:1216-25.

79. GM Knudsen, M Karlsborg, G Thomsen, K Krabbe, L Regeur, T Nygaard, C Videbaek, L Werdelin: **Imaging of dopamine transporters and D2 receptors in patients with Parkinson's disease and multiple system atrophy**. *Eur J Nucl Med Mol Imaging* 2004, **31**:1631-8.

80. B Landwehrmeyer, JM Palacios: **Alterations of neurotransmitter receptors and neurotransmitter transporters in progressive supranuclear palsy**. *J Neural Transm Suppl* 1994, **42**:229-46.

81. R Larisch, W Meyer, A Klimke, F Kehren, H Vosberg, HW Muller-Gartner: **Left-right asymmetry of striatal dopamine D2 receptors**. *Nucl Med Commun* 1998, **19**:781-7.

82. V Laulumaa, JT Kuikka, H Soininen, K Bergstrom, E Lansimies, P Riekkinen: **Imaging of D2 dopamine receptors of patients with Parkinson's disease using single photon emission computed tomography and iodobenzamide I 123**. *Arch Neurol* 1993, **50**:509-12.

83. J Lavalaye, J Booij, L Reneman, JB Habraken, EA van Royen: **Effect of age and gender on dopamine transporter imaging with [123I]FP-CIT SPET in healthy volunteers**. *Eur J Nucl Med* 2000, **27**:867-9.

84. M Lorberboym, R Djaldetti, E Melamed, M Sadeh, Y Lampl: **123I-FP-CIT SPECT imaging of dopamine transporters in patients with cerebrovascular disease and clinical diagnosis of vascular parkinsonism**. *J Nucl Med* 2004, **45**:1688-93.

85. JM Maloteaux, MA Vanisberg, C Laterre, F Javoy-Agid, Y Agid, PM Laduron: **[3H]GBR 12935 binding to dopamine uptake sites: subcellular localization and reduction in Parkinson's disease and progressive supranuclear palsy**. *Eur J Pharmacol* 1988, **156**:331-40.

86. K Marek, R Innis, C van Dyck, B Fussell, M Early, S Eberly, D Oakes, J Seibyl: **[123I]beta-CIT SPECT imaging assessment of the rate of Parkinson's disease progression**. *Neurology* 2001, **57**:2089-94.

87. K Marek, D Jennings, J Seibyl: **Single-photon emission tomography and dopamine transporter imaging in Parkinson's disease**. *Adv Neurol* 2003, **91**:183-91.

88. K Marek, D Jennings, J Seibyl: **Dopamine agonists and Parkinson's disease progression: what can we learn from neuroimaging studies**. *Ann Neurol* 2003, **53 Suppl 3**:S160-6; discussion S166-9.

89. KL Marek, JP Seibyl, SS Zoghbi, Y Zea-Ponce, RM Baldwin, B Fussell, DS Charney, C van Dyck, PB Hoffer, RP Innis: **[123I] beta-CIT/SPECT imaging demonstrates bilateral loss of dopamine transporters in hemi-Parkinson's disease**. *Neurology* 1996, **46**:231-7.

90. PD Mozley, JS Schneider, PD Acton, K Plossl, MB Stern, A Siderowf, NA Leopold, PY Li, A Alavi, HF Kung: **Binding of [99mTc]TRODAT-1 to dopamine transporters in patients with Parkinson's disease and in healthy volunteers**. *J Nucl Med* 2000, **41**:584-9.

91. T Muller, EG Eising, C Reiners, H Przuntek, M Jacob, W Kuhn: **2-[123I]-iodolisuride SPET visualizes dopaminergic loss in de-novo parkinsonian patients: is it a marker of striatal pre-synaptic degeneration?** *Nucl Med Commun* 1997, **18**:1115-21.

92. T Murai, U Muller, K Werheid, D Sorger, M Reuter, T Becker, DY von Cramon, H Barthel: **In vivo evidence for differential association of striatal dopamine and midbrain serotonin systems with neuropsychiatric symptoms in Parkinson's disease**. *J Neuropsychiatry Clin Neurosci* 2001, **13**:222-8.

93. SE Nadeau, MW Couch, CL Devane, SS Shukla: **Regional analysis of D2 dopamine receptors in Parkinson's disease using SPECT and iodine-123-iodobenzamide**. *J Nucl Med* 1995, **36**:384-93.

94. JT O'Brien, S Colloby, J Fenwick, ED Williams, M Firbank, D Burn, D Aarsland, IG McKeith: **Dopamine transporter loss visualized with FP-CIT SPECT in the differential diagnosis of dementia with Lewy bodies**. *Arch Neurol* 2004, **61**:919-25.

95. L Pavics, G Dibo, E Ambrus, T Sera, L Vecsei, L Csernay: **[Initial experiences with 123-Iodine-IBZM neuroreceptor scintigraphy in extrapyramidal disorders]**. *Orv Hetil* 2000, **141**:1073-7.

96. L Pierot, C Desnos, J Blin, R Raisman, D Scherman, F Javoy-Agid, M Ruberg, Y Agid: **D1 and D2-type dopamine receptors in patients with Parkinson's disease and progressive supranuclear palsy**. *J Neurol Sci* 1988, **86**:291-306.

97. MA Piggott, EF Marshall, N Thomas, S Lloyd, JA Court, E Jaros, D Burn, M Johnson, RH Perry, IG McKeith, et al: **Striatal dopaminergic markers in dementia with Lewy bodies, Alzheimer's and Parkinson's diseases: rostrocaudal distribution**. *Brain* 1999, **122 ( Pt 8)**:1449-68.

98. W Pirker: **Correlation of dopamine transporter imaging with parkinsonian motor handicap: how close is it?** *Mov Disord* 2003, **18 Suppl 7**:S43-51.

99. W Pirker, S Asenbaum, M Hauk, S Kandlhofer, J Tauscher, M Willeit, A Neumeister, N Praschak-Rieder, P Angelberger, T Brucke: **Imaging serotonin and dopamine transporters with 123I-beta-CIT SPECT: binding kinetics and effects of normal aging**. *J Nucl Med* 2000, **41**:36-44.

100. W Pirker, I Holler, W Gerschlager, S Asenbaum, G Zettinig, T Brucke: **Measuring the rate of progression of Parkinson's disease over a 5-year period with beta-CIT SPECT**. *Mov Disord* 2003, **18**:1266-72.

101. G Pizzolato, A Cagnin, A Rossato, F Chierichetti, M Fabbri, M Dam, G Ferlin, L Battistin: **Striatal dopamine D2 receptor alterations and response to L-DOPA in Parkinson's disease. A [123I]IBZM SPET study**. *Adv Neurol* 1996, **69**:467-73.

102. G Pizzolato, F Chierichetti, A Rossato, C Briani, M Dam, N Borsato, B Saitta, P Zanco, G Ferlin, L Battistin: **Dopamine receptor SPET imaging in Parkinson's disease: a [123I]-IBZM and [99mTc]-HM-PAO study**. *Eur Neurol* 1993, **33**:143-8.

103. G Pizzolato, F Chierichetti, A Rossato, A Cagnin, M Fabbri, M Dam, G Ferlin, L Battistin: **Alterations of striatal dopamine D2 receptors contribute to deteriorated response to L-dopa in Parkinson's disease: a [123I]-IBZM SPET study**. *J Neural Transm Suppl* 1995, **45**:113-22.

104. M Plotkin, H Amthauer, S Quill, F Marzinzik, F Klostermann, S Klaffke, A Kivi, M Gutberlet, R Felix, A Kupsch: **Imaging of dopamine transporters and D2 receptors in vascular parkinsonism: a report of four cases**. *J Neural Transm* 2005.

105. G Popperl, P Radau, R Linke, K Hahn, K Tatsch: **Diagnostic performance of a 3-D automated quantification method of dopamine D2 receptor SPECT studies in the differential diagnosis of parkinsonism**. *Nucl Med Commun* 2005, **26**:39-43.

106. G Popperl, K Tatsch, E Ruzicka, A Storch, T Gasser, J Schwarz: **Comparison of alpha-dihydroergocryptine and levodopa monotherapy in Parkinson's disease: assessment of changes in DAT binding with [123I]IPT SPECT**. *J Neural Transm* 2004, **111**:1041-52.

107. C Prunier, F Tranquart, JP Cottier, B Giraudeau, S Chalon, D Guilloteau, B De Toffol, F Chossat, A Autret, JC Besnard, et al: **Quantitative analysis of striatal dopamine D2 receptors with 123 I-iodolisuride SPECT in degenerative extrapyramidal diseases**. *Nucl Med Commun* 2001, **22**:1207-14.

108. N Quinn: **A multicenter assessment of dopamine transporter imaging with DOPASCAN/SPECT in parkinsonism**. *Neurology* 2001, **57**:746-7.

109. PE Radau, R Linke, PJ Slomka, K Tatsch: **Optimization of automated quantification of 123I-IBZM uptake in the striatum applied to parkinsonism**. *J Nucl Med* 2000, **41**:220-7.

110. G Ransmayr, K Seppi, E Donnemiller, E Luginger, J Marksteiner, G Riccabona, W Poewe, GK Wenning: **Striatal dopamine transporter function in dementia with Lewy bodies and Parkinson's disease**. *Eur J Nucl Med* 2001, **28**:1523-8.

111. W Reiche, M Grundmann, G Huber: **[Dopamine (D2) receptor SPECT with 123I-iodobenzamide (IBZM) in diagnosis of Parkinson syndrome]**. *Radiologe* 1995, **35**:838-43.

112. T Sasaki, T Amano, J Hashimoto, Y Itoh, K Muramatsu, A Kubo, Y Fukuuchi: **[SPECT imaging using [123I]beta-CIT and [123I]IBF in extrapyramidal diseases]**. *No To Shinkei* 2003, **55**:57-64.

113. HB Saur, P Bartenstein, O Schober, C Oberwittler, H Lerch, H Masur: **[Comparison of D2 receptor scintigraphy (123I-IBZM) with cerebral perfusion (99m-Tc-HMPAO) in extrapyramidal disorders]**. *Nuklearmedizin* 1994, **33**:184-8.

114. C Scherfler, K Seppi, E Donnemiller, G Goebel, C Brenneis, I Virgolini, GK Wenning, W Poewe: **Voxel-wise analysis of [123I]{beta}-CIT SPECT differentiates the Parkinson variant of multiple system atrophy from idiopathic Parkinson's disease**. *Brain* 2005.

115. JB Schulz, T Klockgether, D Petersen, M Jauch, W Muller-Schauenburg, S Spieker, K Voigt, J Dichgans: **Multiple system atrophy: natural history, MRI morphology, and dopamine receptor imaging with 123IBZM-SPECT**. *J Neurol Neurosurg Psychiatry* 1994, **57**:1047-56.

116. J Schwarz, WH Oertel, K Tatsch: **Iodine-123-iodobenzamide binding in parkinsonism: reduction by dopamine agonists but not L-Dopa**. *J Nucl Med* 1996, **37**:1112-5.

117. J Schwarz, A Storch, W Koch, O Pogarell, PE Radau, K Tatsch: **Loss of dopamine transporter binding in Parkinson's disease follows a single exponential rather than linear decline**. *J Nucl Med* 2004, **45**:1694-7.

118. J Schwarz, K Tatsch, G Arnold, T Gasser, C Trenkwalder, CM Kirsch, WH Oertel: **123I-iodobenzamide-SPECT predicts dopaminergic responsiveness in patients with de novo parkinsonism**. *Neurology* 1992, **42**:556-61.

119. J Seibyl, D Jennings, R Tabamo, K Marek: **Neuroimaging trials of Parkinson's disease progression**. *J Neurol* 2004, **251 Suppl 7**:vII9-13.

120. JP Seibyl, M Laruelle, CH van Dyck, E Wallace, RM Baldwin, S Zoghbi, Y Zea-Ponce, JL Neumeyer, DS Charney, PB Hoffer, et al: **Reproducibility of iodine-123-beta-CIT SPECT brain measurement of dopamine transporters**. *J Nucl Med* 1996, **37**:222-8.

121. JP Seibyl, K Marek, K Sheff, RM Baldwin, S Zoghbi, Y Zea-Ponce, DS Charney, CH van Dyck, PB Hoffer, RB Innis: **Test/retest reproducibility of iodine-123-betaCIT SPECT brain measurement of dopamine transporters in Parkinson's patients**. *J Nucl Med* 1997, **38**:1453-9.

122. JP Seibyl, K Marek, K Sheff, S Zoghbi, RM Baldwin, DS Charney, CH van Dyck, RB Innis: **Iodine-123-beta-CIT and iodine-123-FPCIT SPECT measurement of dopamine transporters in healthy subjects and Parkinson's patients**. *J Nucl Med* 1998, **39**:1500-8.

123. JP Seibyl, KL Marek, D Quinlan, K Sheff, S Zoghbi, Y Zea-Ponce, RM Baldwin, B Fussell, EO Smith, DS Charney, et al: **Decreased single-photon emission computed tomographic [123I]beta-CIT striatal uptake correlates with symptom severity in Parkinson's disease**. *Ann Neurol* 1995, **38**:589-98.

124. H Sjoholm, SI Mellgren, J Sundsfjord: **Nigro-striatal degeneration demonstrated in parkinsonian patients with iodine-123-beta-CIT SPECT: methods of quantitation**. *Acta Neurol Scand* 1997, **96**:91-6.

125. M Sohmiya, M Tanaka, Y Aihara, K Okamoto: **Structural changes in the midbrain with aging and Parkinson's disease: an MRI study**. *Neurobiol Aging* 2004, **25**:449-53.

126. U Sommer, T Hummel, K Cormann, A Mueller, J Frasnelli, J Kropp, H Reichmann: **Detection of presymptomatic Parkinson's disease: combining smell tests, transcranial sonography, and SPECT**. *Mov Disord* 2004, **19**:1196-202.

127. W Staffen, N Hondl, E Trinka, R Zenzmaier, G Ladurner: **SPET investigations in extrapyramidal diseases using specific ligands**. *Nucl Med Commun* 1997, **18**:159-63.

128. W Staffen, A Mair, J Unterrainer, E Trinka, C Bsteh, G Ladurner: **[123I] beta-CIT binding and SPET compared with clinical diagnosis in parkinsonism**. *Nucl Med Commun* 2000, **21**:417-24.

129. W Staffen, A Mair, J Unterrainer, E Trinka, G Ladurner: **Measuring the progression of idiopathic Parkinson's disease with [123I] beta-CIT SPECT**. *J Neural Transm* 2000, **107**:543-52.

130. JK Staley, S Krishnan-Sarin, S Zoghbi, G Tamagnan, M Fujita, JP Seibyl, PK Maciejewski, S O'Malley, RB Innis: **Sex differences in [123I]beta-CIT SPECT measures of dopamine and serotonin transporter availability in healthy smokers and nonsmokers**. *Synapse* 2001, **41**:275-84.

131. HL Stockbridge, D Lewis, B Eisenberg, M Lee, S Schacher, G van Belle, M Keifer, CA Brodkin, D Buchwald: **Brain SPECT: a controlled, blinded assessment of intra-reader and inter-reader agreement**. *Nucl Med Commun* 2002, **23**:537-44.

132. RL Swanson, AB Newberg, PD Acton, A Siderowf, N Wintering, A Alavi, PD Mozley, K Plossl, M Udeshi, H Hurtig: **Differences in [99mTc]TRODAT-1 SPECT binding to dopamine transporters in patients with multiple system atrophy and Parkinson's disease**. *Eur J Nucl Med Mol Imaging* 2005, **32**:302-7.

133. K Tatsch, J Schwarz, PD Mozley, R Linke, O Pogarell, WH Oertel, RS Fieber, K Hahn, HF Kung: **Relationship between clinical features of Parkinson's disease and presynaptic dopamine transporter binding assessed with [123I]IPT and single-photon emission tomography**. *Eur J Nucl Med* 1997, **24**:415-21.

134. H Terashi, K Nagata, Y Hirata, J Hatazawa, H Utsumi: **[Study on dopamine D2 binding capacity in vascular parkinsonism]**. *Rinsho Shinkeigaku* 2001, **41**:659-64.

135. S Thobois, M Jahanshahi, S Pinto, R Frackowiak, P Limousin-Dowsey: **PET and SPECT functional imaging studies in Parkinsonian syndromes: from the lesion to its consequences**. *Neuroimage* 2004, **23**:1-16.

136. G Tissingh, P Bergmans, J Booij, A Winogrodzka, JC Stoof, EC Wolters, EA Van Royen: **[123I]beta-CIT single-photon emission tomography in Parkinson's disease reveals a smaller decline in dopamine transporters with age than in controls**. *Eur J Nucl Med* 1997, **24**:1171-4.

137. G Tissingh, P Bergmans, J Booij, A Winogrodzka, EA van Royen, JC Stoof, EC Wolters: **Drug-naive patients with Parkinson's disease in Hoehn and Yahr stages I and II show a bilateral decrease in striatal dopamine transporters as revealed by [123I]beta-CIT SPECT**. *J Neurol* 1998, **245**:14-20.

138. G Tissingh, J Booij, P Bergmans, A Winogrodzka, AG Janssen, EA van Royen, JC Stoof, EC Wolters: **Iodine-123-N-omega-fluoropropyl-2beta-carbomethoxy-3beta-(4-iod ophenyl)tropane SPECT in healthy controls and early-stage, drug-naive Parkinson's disease**. *J Nucl Med* 1998, **39**:1143-8.

139. K Torizuka, Y Mizuno, A Kubo, J Konishi, Y Yonekura, J Hatazawa, T Momose, M Murata, T Amano, H Fukuyama, et al: **[Phase 2 clinical study of 123I-IBF, a dopamine D2 receptor imaging agent, to evaluate clinical efficacy and safety in Parkinson's disease and Parkinson syndromes]**. *Kaku Igaku* 1999, **36**:845-64.

140. F Tranquart, FH Le Bras, B de Toffol, A Autret, D Guilloteau, JL Baulieu: **[Progressive supranuclear paralysis. Quantification of dopamine D2 receptors using radionuclide tomography]**. *Presse Med* 1994, **23**:1299-300.

141. T Tsuchida, JR Ballinger, D Vines, YJ Kim, K Utsunomiya, AE Lang, M Ichise: **Reproducibility of dopamine transporter density measured with 123I-FPCIT SPECT in normal control and Parkinson's disease patients**. *Ann Nucl Med* 2004, **18**:609-16.

142. KY Tzen, CS Lu, TC Yen, SP Wey, G Ting: **Differential diagnosis of Parkinson's disease and vascular parkinsonism by (99m)Tc-TRODAT-1**. *J Nucl Med* 2001, **42**:408-13.

143. J Vaamonde, R Ibanez, AM Garcia, V Poblete: **[Study of the pre and post-synaptic dopaminergic system by DaTSCAN/IBZM SPECT in the differential diagnosis of parkinsonism in 75 patients]**. *Neurologia* 2004, **19**:292-300.

144. CH van Dyck, JP Seibyl, RT Malison, M Laruelle, SS Zoghbi, RM Baldwin, RB Innis: **Age-related decline in dopamine transporters: analysis of striatal subregions, nonlinear effects, and hemispheric asymmetries**. *Am J Geriatr Psychiatry* 2002, **10**:36-43.

145. K Van Laere, P Santens, T Bosman, J De Reuck, L Mortelmans, R Dierckx: **Statistical parametric mapping of (99m)Tc-ECD SPECT in idiopathic Parkinson's disease and multiple system atrophy with predominant parkinsonian features: correlation with clinical parameters**. *J Nucl Med* 2004, **45**:933-42.

146. A Varrone, KL Marek, D Jennings, RB Innis, JP Seibyl: **[(123)I]beta-CIT SPECT imaging demonstrates reduced density of striatal dopamine transporters in Parkinson's disease and multiple system atrophy**. *Mov Disord* 2001, **16**:1023-32.

147. NP Verhoeff, O Kapucu, E Sokole-Busemann, EA van Royen, AG Janssen: **Estimation of dopamine D2 receptor binding potential in the striatum with iodine-123-IBZM SPECT: technical and interobserver variability**. *J Nucl Med* 1993, **34**:2076-84.

148. RJ Vermeulen, EC Wolters, G Tissingh, J Booij, AG Janssen, J Habraken, E Sokole-Busemann, JC Stoof, EA Van Royen: **Evaluation of [123I] beta-CIT binding with SPECT in controls, early and late Parkinson's disease**. *Nucl Med Biol* 1995, **22**:985-91.

149. YH Weng, TC Yen, MC Chen, PF Kao, KY Tzen, RS Chen, SP Wey, G Ting, CS Lu: **Sensitivity and specificity of 99mTc-TRODAT-1 SPECT imaging in differentiating patients with idiopathic Parkinson's disease from healthy subjects**. *J Nucl Med* 2004, **45**:393-401.

150. GK Wenning, E Donnemiller, R Granata, G Riccabona, W Poewe: **123I-beta-CIT and 123I-IBZM-SPECT scanning in levodopa-naive Parkinson's disease**. *Mov Disord* 1998, **13**:438-45.

151. A Winogrodzka, P Bergmans, J Booij, EA van Royen, AG Janssen, EC Wolters: **[123I]FP-CIT SPECT is a useful method to monitor the rate of dopaminergic degeneration in early-stage Parkinson's disease**. *J Neural Transm* 2001, **108**:1011-9.

152. A Winogrodzka, P Bergmans, J Booij, EA van Royen, JC Stoof, EC Wolters: **[(123)I]beta-CIT SPECT is a useful method for monitoring dopaminergic degeneration in early stage Parkinson's disease**. *J Neurol Neurosurg Psychiatry* 2003, **74**:294-8.
