# Supplementary material for: Meta-analysis of the literature on diagnostic accuracy of SPECT in parkinsonian syndromes
Source: BMC Neurol. 2007 Sep 1;7:27. doi: 10.1186/1471-2377-7-27 (PMC2064928; doi:10.1186/1471-2377-7-27)
Supplement: Additional file 3 — reference list 2. reference list of the studies included in the meta-analysis [file 1471-2377-7-27-S3.doc]

[1-33]

1. **A multicenter assessment of dopamine transporter imaging with DOPASCAN/SPECT in parkinsonism. Parkinson Study Group**. *Neurology* 2000, **55**:1540-7.

2. S Asenbaum, W Pirker, P Angelberger, G Bencsits, M Pruckmayer, T Brucke: **[123I]beta-CIT and SPECT in essential tremor and Parkinson's disease**. *J Neural Transm* 1998, **105**:1213-28.

3. TS Benamer, J Patterson, DG Grosset, J Booij, K de Bruin, E van Royen, JD Speelman, MH Horstink, HJ Sips, RA Dierckx, et al: **Accurate differentiation of parkinsonism and essential tremor using visual assessment of [123I]-FP-CIT SPECT imaging: the [123I]-FP-CIT study group**. *Mov Disord* 2000, **15**:503-10.

4. J Booij, JD Speelman, MW Horstink, EC Wolters: **The clinical benefit of imaging striatal dopamine transporters with [123I]FP-CIT SPET in differentiating patients with presynaptic parkinsonism from those with other forms of parkinsonism**. *Eur J Nucl Med* 2001, **28**:266-72.

5. A Buck, G Westera, M Sutter, C Albani, HF Kung, GK vonSchulthess: **Iodine-123-IBF SPECT evaluation of extrapyramidal diseases**. *J Nucl Med* 1995, **36**:1196-200.

6. J Eerola, PJ Tienari, S Kaakkola, P Nikkinen, J Launes: **How useful is [123I]beta-CIT SPECT in clinical practice?** *J Neurol Neurosurg Psychiatry* 2005, **76**:1211-6.

7. W Gerschlager, G Bencsits, W Pirker, BR Bloem, S Asenbaum, D Prayer, JC Zijlmans, M Hoffmann, T Brucke: **[123I]beta-CIT SPECT distinguishes vascular parkinsonism from Parkinson's disease**. *Mov Disord* 2002, **17**:518-23.

8. TH Haapaniemi, A Ahonen, P Torniainen, KA Sotaniemi, VV Myllyla: **[123I]beta-CIT SPECT demonstrates decreased brain dopamine and serotonin transporter levels in untreated parkinsonian patients**. *Mov Disord* 2001, **16**:124-30.

9. WS Huang, SZ Lin, JC Lin, SP Wey, G Ting, RS Liu: **Evaluation of early-stage Parkinson's disease with 99mTc-TRODAT-1 imaging**. *J Nucl Med* 2001, **42**:1303-8.

10. YJ Kim, M Ichise, JR Ballinger, D Vines, SS Erami, T Tatschida, AE Lang: **Combination of dopamine transporter and D2 receptor SPECT in the diagnostic evaluation of PD, MSA, and PSP**. *Mov Disord* 2002, **17**:303-12.

11. MS Lee, YD Kim, JH Im, HJ Kim, JO Rinne, KP Bhatia: **123I-IPT brain SPECT study in essential tremor and Parkinson's disease**. *Neurology* 1999, **52**:1422-6.

12. A Lokkegaard, LM Werdelin, L Friberg: **Clinical impact of diagnostic SPET investigations with a dopamine re-uptake ligand**. *Eur J Nucl Med Mol Imaging* 2002, **29**:1623-9.

13. CS Lu, YH Weng, MC Chen, RS Chen, KY Tzen, SP Wey, G Ting, HC Chang, TC Yen: **99mTc-TRODAT-1 imaging of multiple system atrophy**. *J Nucl Med* 2004, **45**:49-55.

14. C Messa, MA Volonte, F Fazio, F Zito, A Carpinelli, A d'Amico, G Rizzo, RM Moresco, E Paulesu, M Franceschi, et al: **Differential distribution of striatal [123I]beta-CIT in Parkinson's disease and progressive supranuclear palsy, evaluated with single-photon emission tomography**. *Eur J Nucl Med* 1998, **25**:1270-6.

15. T Muller, J Farahati, W Kuhn, EG Eising, H Przuntek, C Reiners, HH Coenen: **[123I]beta-CIT SPECT visualizes dopamine transporter loss in de novo parkinsonian patients**. *Eur Neurol* 1998, **39**:44-8.

16. WH Oertel, J Schwarz, K Tatsch, G Arnold, T Gasser, CM Kirsch: **IBZM-SPECT as predictor for dopamimetic responsiveness of patients with de novo parkinsonian syndrome**. *Adv Neurol* 1993, **60**:519-24.

17. C Oyanagi, Y Katsumi, T Hanakawa, T Hayashi, DD Thuy, K Hashikawa, Y Nagahama, H Fukuyama, H Shibasaki: **Comparison of striatal dopamine D2 receptors in Parkinson's disease and progressive supranuclear palsy patients using [123I] iodobenzofuran single-photon emission computed tomography**. *J Neuroimaging* 2002, **12**:316-24.

18. W Pirker, S Asenbaum, G Bencsits, D Prayer, W Gerschlager, L Deecke, T Brucke: **[123I]beta-CIT SPECT in multiple system atrophy, progressive supranuclear palsy, and corticobasal degeneration**. *Mov Disord* 2000, **15**:1158-67.

19. W Pirker, S Asenbaum, S Wenger, J Kornhuber, P Angelberger, L Deecke, I Podreka, T Brucke: **Iodine-123-epidepride-SPECT: studies in Parkinson's disease, multiple system atrophy and Huntington's disease**. *J Nucl Med* 1997, **38**:1711-7.

20. W Pirker, S Djamshidian, S Asenbaum, W Gerschlager, G Tribl, M Hoffmann, T Brucke: **Progression of dopaminergic degeneration in Parkinson's disease and atypical parkinsonism: a longitudinal beta-CIT SPECT study**. *Mov Disord* 2002, **17**:45-53.

21. M Plotkin, H Amthauer, S Klaffke, A Kuhn, L Ludemann, G Arnold, KD Wernecke, A Kupsch, R Felix, S Venz: **Combined (123)I-FP-CIT and (123)I-IBZM SPECT for the diagnosis of parkinsonian syndromes: study on 72 patients**. *J Neural Transm* 2005, **112**:677-92.

22. L Schelosky, J Hierholzer, J Wissel, M Cordes, W Poewe: **Correlation of clinical response in apomorphine test with D2-receptor status as demonstrated by 123I IBZM-SPECT**. *Mov Disord* 1993, **8**:453-8.

23. J Schwarz, A Antonini, K Tatsch, CM Kirsch, WH Oertel, KL Leenders: **Comparison of 123I-IBZM SPECT and 11C-raclopride PET findings in patients with parkinsonism**. *Nucl Med Commun* 1994, **15**:806-13.

24. J Schwarz, R Linke, M Kerner, PD Mozley, C Trenkwalder, T Gasser, K Tatsch: **Striatal dopamine transporter binding assessed by [I-123]IPT and single photon emission computed tomography in patients with early Parkinson's disease: implications for a preclinical diagnosis**. *Arch Neurol* 2000, **57**:205-8.

25. J Schwarz, K Tatsch, G Arnold, M Ott, C Trenkwalder, CM Kirsch, WH Oertel: **123I-iodobenzamide-SPECT in 83 patients with de novo parkinsonism**. *Neurology* 1993, **43**:S17-20.

26. J Schwarz, K Tatsch, T Gasser, G Arnold, WH Oertel: **[123]IBZM binding predicts dopaminergic responsiveness in patients with parkinsonism and previous dopaminomimetic therapy**. *Mov Disord* 1997, **12**:898-902.

27. J Schwarz, K Tatsch, T Gasser, G Arnold, O Pogarell, G Kunig, WH Oertel: **123I-IBZM binding compared with long-term clinical follow up in patients with de novo parkinsonism**. *Mov Disord* 1998, **13**:16-9.

28. K Seppi, MF Schocke, E Donnemiller, R Esterhammer, C Kremser, C Scherfler, A Diem, W Jaschke, GK Wenning, W Poewe: **Comparison of diffusion-weighted imaging and [123I]IBZM-SPECT for the differentiation of patients with the Parkinson variant of multiple system atrophy from those with Parkinson's disease**. *Mov Disord* 2004, **19**:1438-45.

29. D Stoffers, J Booij, L Bosscher, A Winogrodzka, EC Wolters, HW Berendse: **Early-stage [(123)I]beta-CIT SPECT and long-term clinical follow-up in patients with an initial diagnosis of Parkinson's disease**. *Eur J Nucl Med Mol Imaging* 2005.

30. K Tatsch, J Schwarz, WH Oertel, CM Kirsch: **SPECT imaging of dopamine D2 receptors with 123I-IBZM: initial experience in controls and patients with Parkinson's syndrome and Wilson's disease**. *Nucl Med Commun* 1991, **12**:699-707.

31. K Van Laere, L De Ceuninck, R Dom, J Van den Eynden, H Vanbilloen, J Cleynhens, P Dupont, G Bormans, A Verbruggen, L Mortelmans: **Dopamine transporter SPECT using fast kinetic ligands: 123I-FP-beta-CIT versus 99mTc-TRODAT-1**. *Eur J Nucl Med Mol Imaging* 2004, **31**:1119-27.

32. E van Royen, NF Verhoeff, JD Speelman, EC Wolters, MA Kuiper, AG Janssen: **Multiple system atrophy and progressive supranuclear palsy. Diminished striatal D2 dopamine receptor activity demonstrated by 123I-IBZM single photon emission computed tomography**. *Arch Neurol* 1993, **50**:513-6.

33. AMM Vlaar, AGH Kessels, T Nijs de, vMJPG Kroonenburgh, WEJ Weber: **Diagnostic value of Single Photon Computer Tomography to differentiate patients with**

**Idiopathic Parkinson Disease from patients with other parkinsonian syndromes. Using**

**the clinical diagnosis after follow-up as golden standard.** *submitted* 2006.
